# Effect of Annealing Conditions of High-Energy Ball-Milled Sm(Fe, Co, Ti)_12_ Alloys Doped with Zr on Microstructure and Magnetic Properties

**DOI:** 10.3390/ma18071642

**Published:** 2025-04-03

**Authors:** Margarit Gjoka, Charalampos Sarafidis, Dimitrios Niarchos, George Hadjipanayis

**Affiliations:** 1 Institute of Nanoscience and Nanotechnology, National Centre for Science and Research “Demokritos”, 15341 Athens, Greece; 2 Department of and Physics, School of Sciences, Aristotle University of Thessaloniki, 54124 Thessaloniki, Greece; hsara@physics.auth.gr; 3 Department of Physics, University of Delaware, Newark, NJ 19716, USA; 4 Department of Chemical Engineering, Northeastern University, Boston, MA 02115, USA

**Keywords:** rare earth permanent magnets, ball-milling process, SmFe_12_-based alloys, crystal structure

## Abstract

The tetragonal R_1−x_Zr_x_(FeCo)_11_Ti alloys, where R is a rare earth and Ti a transition metal, are promising candidates for permanent magnets. Sm_1−x_Zr_x_(Fe_0.8_Co_0.2_)_12−y_Ti_y_ (x = 0 and 0.25; y = 1 and 0.7) master alloys were prepared by arc melting under argon atmosphere. Some of the samples were almost single-phase compounds at 1:12, with a very small amount of a-Fe(Co). Partially replacing Sm with Zr produced alloys with small amounts of Sm(FeCo)_2_ Laves-type phases. The as-cast ingots were milled using high-energy ball milling (HEBM) for different times in an argon atmosphere and then annealed at 973 K–1173 K at different interval times (15–90 min). After annealing, the sample milled for 4 h contained a large variation of grain size from 2–4 μm to 20 μm or larger, while, after annealing, the other sampled milled for 8 h exhibited grains size in the range of 2–6 μm; therefore, their coercivity was higher, reaching a maximum value of 5.5 kOe for SmFe_9_Co_2_Ti annealed at 1123 K for 60 min. Coercivity was strongly affected by the annealing temperature and time. The microstructure evolution with emphasis on the particles size during annealing and their correlation with coercivity are herein discussed.

## 1. Introduction

The challenging, world-wide environmental problems are the main driving factor of a series of important technological advances. Sustainable transportation and effective electricity production like offshore wind generators have skyrocketed the demand for materials suitable for permanent magnets (PM). Extensive demand induces challenges in production and supply channels, making necessary the development of new materials, especially with low content in critical raw metals like the rare earths (RE) or other important metals, like Co [1,2,3,4,5].

The tetragonal ThMn_12_-type alloys with nominal composition R(Fe, T)_12_, where R is a rare earth atom and T is a transition metal atom, are excellent candidates for permanent magnets due to their high Curie temperature, large magnetization strength, and strong uniaxial magneto-crystalline anisotropy. Their magnetization and intrinsic anisotropy outperform well-established magnetic materials, and their Curie temperatures are high at close to 600 K, establishing a working temperature higher than that of Nd_2_Fe_14_B without the need for doping with heavy RE atoms [6,7,8]. Coercivity values above 10 kOe (μ_0_H 1 T) have been observed [9]. All these excellent properties are combined with the highest T/R ratio as compared to other magnetic families of intermetallic compounds, and this explains why these alloys have been the focus of scientific research in recent years [10,11].

It is known that the enthalpy (or energy) of the formation of a compound is the energy associated with the reaction of forming the compound from its constituent elements. The formation energy of SmFe_12_ in the ground state is positive, with a value about 0.0193 eV/atom; as a result of which, it is more thermodynamically unstable than the pure elements Sm and Fe corresponding to the same composition. Theoretical and experimental research indicates that the addition of small amounts of alloying elements T such as Ti, Mo, V, Al, Si, and Ga leads to negative formation energies of RFe_12−x_T_x_ alloys, stabilizing the tetragonal structure, with Ti being the most favored element [12]. However, these additions result in a decline in the magnetic properties [12,13,14,15,16,17,18,19]. This has led to investigations for achieving minimal use of Ti in SmFe_12_-based compounds, such as those reported in [16,20]. Another prominent path is by replacing Fe with Co. It is well known that this specific substitution affects both stability and the magnetic properties of the R-T intermetallics, so it may have the potential to stabilize the tetragonal 1:12 phase. The 3D magnetocrystalline anisotropy constant remains uniaxial for the relatively small Co replacement for Fe and at room temperature K_1_ reaches about 0.9 ΜJm^−3^ [19]. With up to 20% Co replacement for Fe, both the nature of anisotropy and the anisotropy field are not affected significantly. At the same time, the magnetization is improved. The maximum magnetization of the theoretical compound SmFe_12_ could be as high as 160 Am^2^/kg; however, this alloy does not exist in bulk form [20].

In an early survey of SmTiFe_11−x_Co_x_ alloys reported by S.F. Cheng et al. [21], it was found that the tetragonal phase could be stabilized for x ≤ 2 while maintaining the easy magnetization direction along the c-axis. This is important since it is very often that in these systems, Co favors a different type of magnetocrystalline anisotropy. For replacement beyond x = 2, other phases such as 2:17 usually appear, while the magnetic moments are canted in a conical arrangement [21]. Hirayama et al. reported that ThMn_12_-type structure can also be stabilized by Co addition in epitaxially grown thin films with nominal composition Sm(Fe_0.8_Co_0.2_)_12_ [22]. Furthermore, they found that the intrinsic hard magnetic properties were superior even to those of Nd_2_Fe_14_B with a Curie temperature of 859 K, an anisotropy field of 1.2 T, and a spontaneous magnetization of 1.78 T. There are also many reports on Sm(FeT)_12_ (T = Ti, Mo, V, Al, Si, Ga) alloys and their ribbons produced by melt-spinning or strip flakes as well as their isotropic powders produced by rapid solidification or mechanical alloying [9,11,13,14,15,23,24,25,26]. Because of their low remanent magnetization, these isotropic powders need more improvements in processing in order to be used as a basis for PM applications. There are also reports on nanocomposite powders for Sm(FeCoT)_12_ alloys. Additionally, the effect of particle size on the thermal decomposition of powders with nominal composition Sm(Fe_0.8_Co_0.2_)_11_Ti, Sm(Fe_0.8_Co_0.2_)_10_VTi, Sm(Fe_0.8_Co_0.2_)_10.5_Ga_0.5_Ti, and Sm(Fe_0.8_Co_0.2_)_10.5_Cu_0.5_Ti has been studied. These materials were prepared by jet milling, and they were subsequently annealed in various temperatures. Additionally, the case of annealed Sm_8_Fe_73.5_Ti_8_V_8_Ga_0.5_Al_2_ alloy has been also reported. The latter was pulverized using the nitrogen jet-milling process, obtaining particles with an average size of 2–5 µm, followed by a sintering process. In these works, the importance of the type of stabilizing T element as well as its concentration effects on the properties of the materials were also outlined [26,27].

A very interesting result was observed after partial substitution of Sm by Zr. This modification can improve the magnetic properties under certain conditions. Zr is known for its ability to substitute for both R or T atoms in similar R-T intermetallic compounds. In ThMn_12_-based materials, the partial substitution of Sm for Zr has a positive effect on the stabilization of Sm(FeCo)_12_ alloys [20]. Surprisingly, the saturation magnetization increases further; for the case of Sm_0.74_Zr_0.26_(Fe_0.8_Co_0.2_)_12_, compounds can reach a value of μ_0_Ms = 1.9 T [20,28,29]. This specific value is the highest ever achieved in R-T intermetallic compounds. Yao et al. measured a slightly lower magnetization at about μ_0_Ms = 1.4 T in ribbons of nominal composition Sm_0.74_Zr_0.26_(Fe_0.8_Co_0.2_)_12_. This μ_0_Ms value was combined with both very high anisotropy constants (K_1_ = 3.04 MJm^−3^, K_2_ = 0.57 MJm^−3^) and an increased Curie temperature close to 675 K [30].

The partial substitution of Sm by Zr has been further exploited in the literature. Rapidly quenched materials produced by either by melt-spinning technique with nominal stoichiometry (SmR)(FeCo)_12−x_Ti_x_ (R = Y, Zr) or strip-casting method of (R, Zr)(Fe, Co)_12−x_Ti_x_ (R = Nd and Sm) alloys [31,32,33] have also been used to produce materials with interesting magnetic properties. The advancement of computational tools has also led to relevant contributions where Zr was also found to positively affect magnetization, as in other previously reported cases [28,34,35,36]. Additionally, the thermodynamic stability of the Fe rich compounds can be improved using the multi-element alloying approach, which can also minimize the degradation of magnetic properties [20,33,37,38].

Following this specific improvement method, a few years ago, we presented preliminary results of high-energy ball-milled (HEBM) of Sm_1−x_Zr_x_(Fe_0.8_Co_0.2_)_12−y_Ti_y_ (x = 0 and 0.25; y = 1 and 0.7) materials [39]. Partial substitution of Sm with Zr significantly improved the properties of these compounds and their potential for applications as PM [11]. While Ti is a very effective element for thermodynamically stabilizing the tetragonal structure, it has the drawback that it degrades the magnetic properties [12]. Introduction of Zr may assist in stabilizing the structure while reducing Ti content [31]. Kobayashi et al. explained this stabilizing effect by examining the average Fe–Fe interatomic distances at Fe(8i), Fe(8j), and Fe(8f) sites. They observed that the iron atoms in 8i positions are arranged in a distance much greater than their atomic radii; larger Ti atoms may provide a way to accommodate the interatomic distance and make metallic hybridization possible. Incorporation of Zr, which is smaller than Sm, may assist in this direction [33].

It is evident that this specific system has large potential for applications. In the present study, we extend our previous work by providing a wider view of HEBM powders of Sm(Fe, Co, Ti)_12_ alloys doped with Zr, focusing on the effect of annealing conditions of powders on microstructure and magnetic properties.

## 2. Materials and Methods

Sm_1−x_Zr_x_(FeCo)_12−y_Ti_y_ (x = 0 and 0.25; y = 1 and 0.7) alloys were prepared by arc melting in a high-purity argon atmosphere and with excess Sm content to compensate for evaporation during melting. The nominal composition of the first two series of alloys were SmFe_9_Co_2_Ti (A-series) and SmFe_9.04_Co_2.26_Ti_0.7_ (B-series), respectively. For the other two alloys, we partially substituted Zr for Sm, and the nominal composition was Sm_0.75_Zr_0.25_Fe_9_Co_2_Ti (C-series) and Sm_0.75_Zr_0.25_Fe_9.04_Co_2.26_Ti_0.7_ (D-series). Parts from all sample series were mechanically treated in a SPEX-8000 high-energy ball-milling device (HEBM) with a frequency of 875 cycles per minute for different milling times (4 and 8 h). The experiments were performed in a hardened steel vial with a diameter of 5.0 cm and a cylindrical length of 6.5 cm using stainless-steel balls, and the powders were embedded in ethanol. A combination of balls with diameters of 12 mm (3 balls), 8 mm (5 balls), 5.5 mm (12 balls), and 4 mm (12 balls) were used, with a total mass of 42.84 gr. The ball-to-powder weight ratio was 10:1. All samples were subsequently annealed at different times (15–90 min) and temperatures (973 K–1173 K) in order to recrystallize the 1:12 phase and achieve improved coercivity.

X-ray diffraction (XRD) data of powder samples were obtained using a SIEMENS D500 diffractometer (Munich, Germany) with Cu-Kα radiation in the range 2θ = 20–90 degrees 2θ and step 0.03°/5 s. The magnetic properties of the alloys were determined by thermomagnetic analysis (TMA) and a vibrating sample magnetometer (VSM). Thermomagnetic analysis data were obtained under an external field of about 0.015 T (μ_0_H) using small pieces. Magnetization measurements were also performed at room temperature on magnetically oriented powders fixed in epoxy resin under an applied field up to 3 T (μ_0_H). XRD measurements on magnetically aligned powders were also carried out to study the magnetocrystalline anisotropy of the materials. Particle sizes and their distribution was estimated using scanning electron microscopy (SEM) (PHENOM PRO-X, Rotterdam, The Netherlands).

## 3. Results and Discussion

### 3.1. Structure of Arc-Melting Alloys

**Figure 1:**
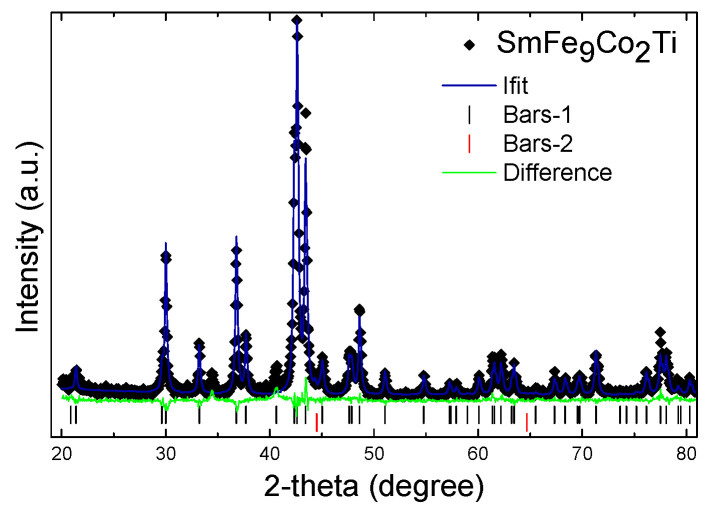
XRD spectrum of arc-melted SmFe_9_Co_2_Ti alloy refined by Rietveld method. (⬪) denotes experimental points. The continuous line corresponds to the calculated spectrum. Vertical bars (I) and (II) at the bottom indicate the position of the Bragg peaks for 1:12 phase and α-(Fe, Co), respectively. The continuous line at the bottom is the difference between the experimental intensity values and the calculated one.

According to the Rietveld analysis, two of the as-cast samples with an atomic content of Ti equal to 1 (samples A and C) were almost single-phase, with the tetragonal 1:12 ThMn_12_-type structure (No 139, Space Group I4/mmm). Ti and Co atoms were assumed to be arranged at 8i and 8f sites, respectively. In Figure 1, the corresponding X-ray diffraction plot is presented for the sample A. The unit cell parameters **a** and **c** for 1:12 phase are 0.8557 and 0.4784 nm, respectively. Impurity α-(Fe, Co) phase appeared with minor content, maximum 1.24 wt.%, and a unit cell parameter a = 0.2885 nm, slightly larger than the corresponding value of α-Fe.

**Figure 2:**
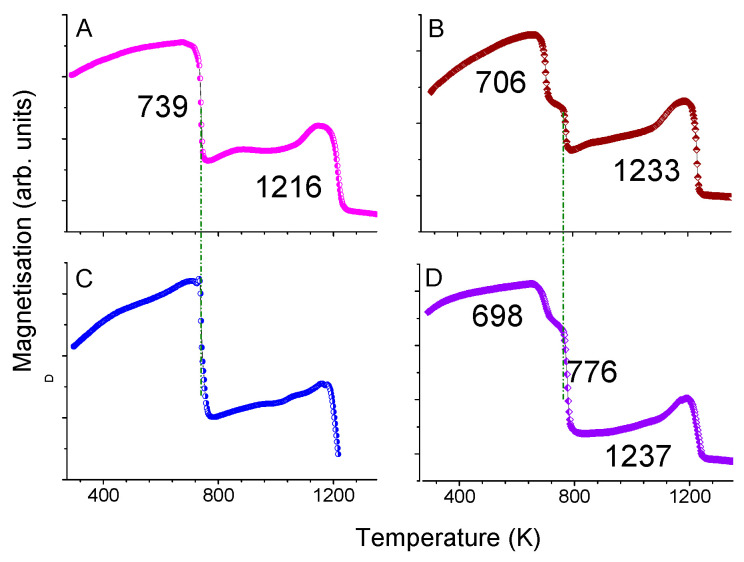
Thermomagnetic curves of as-cast alloys SmFe_9_Co_2_Ti (**A**), Sm_0.75_Zr_0.25_Fe_9_Co_2_Ti (**B**), Sm Fe_9.04_Co_2.26_Ti_0.7_ (**C**), and Sm_0.75_Zr_0.25_Fe_9.04_Co_2.26_Ti_0.7_ (**D**).

In Figure 2, the thermomagnetic curves of all as-casted alloys are presented. The curves of the samples A and C exhibit two magnetic transitions at 739(5) and 1216(5) K for the 1:12 and α-(Fe, Co) phases, respectively. The apparent high-magnetic transition of α-(Fe, Co) is a result of the 1:12 phase. The Curie transitions of α-(Fe, Co), depending on the amount of both Fe and Co, are usually in the range 1193–1258 K [40].

When Sm was partially substituted by Zr, a third phase Sm(FeCo)_2_ of ZnMg_2_ Laves-type appeared (samples B and D) for both cases independently of Ti content; Ti content equaled either 1 or 0.7. The Curie temperatures of these additional phases are 706(5) and 698(5) K for the samples B and D, respectively. It is worth mentioning that the Curie temperature for SmFe_2_ compound is 700 K [41]. The Curie temperature for the 1:12 phase is 776 K for both samples B and D, close to that reported before in the literature [20,21,33,36]. Zr has a negative effect on the Curie temperature, but the increase in Co amount compensates for this, and the compound retains a relatively large temperature endurance, as in the case of sample D. The magnetic transitions of the minor α-(Fe, Co) phase were detected at 1233(5) and 1237(5) K for the samples B and D, respectively (Figure 2), probably related to minor deviations of the Co content in α-(Fe, Co) minority phase.

#### Structure and Magnetic Properties of High-Energy Ball Milling (HEBM)

**Figure 3:**
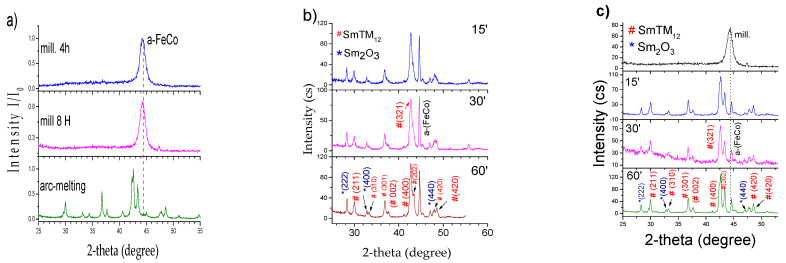
(**a**) XRD of SmFe_9_Co_2_Ti powders milled for 4 h and 8 h, respectively; (**b**) XRD diagrams of annealed powder of SmFe_9_Co_2_Ti milled for 4 h at 1073 K 15, 30, and 60 min and the respective Miler’s indexes of 1:12 and Sm_2_O_3_; (**c**) XRD diagrams of annealed powder of SmFe_9_Co_2_Ti milled for 4 h at 1123 K 15, 30, and 60 min.

The as-cast ingots were milled using high-energy ball milling (HEBM) for different milling times, namely 4 and 8 h in Ar atmosphere, maintaining a mass ratio of balls to powder 10:1. All samples after milling exhibited an amorphous/disordered phase, as it was shown in Figure 3a for SmFe_9_Co_2_Ti. Only a broad reflection appeared at the area of 44 degrees 2θ, which belongs to the α-(Fe, Co) partially crystallized into larger grains.

After thermal treatment at temperatures ranging from 973 to 1173 K for different periods of time (15–90 min), the amorphous powders recrystallized in a 1:12 majority phase, however, with slightly larger α-(Fe, Co) content. A small amount of Sm_2_O_3_ oxide was formed, and the respective Milers indexes are shown in the Figure 3b. The possibility of the appearance of other phases like Sm(FeCo)_2_ with MgZn_2_ structure and 2:17 cannot be excluded due to the similarity in the relevant XRD patterns; however, their respective reflections are barely seen. Typical X-ray diffraction patterns for sample A milled for 4 h and annealed at the range 15–60 min are presented in the Figure 3b.

**Figure 4:**
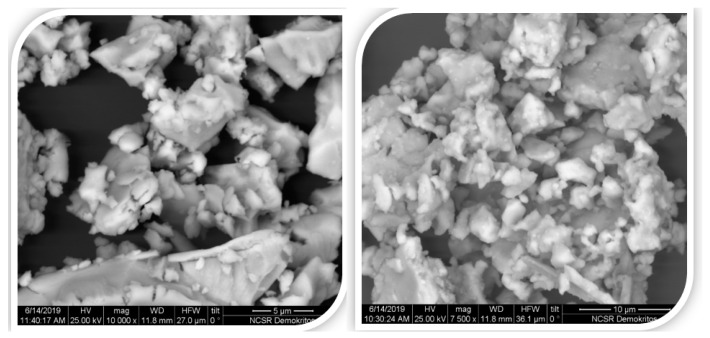
SEM images of SmFe_9_Co_2_Ti powder, milled for 4 h (on the **left**) and 8 h (on the **right**) and then annealed at 1123 K.

Optimal grains sizes for rare earth permanents magnets are reported by many researchers to be close to the range 2–6 μm. W.F. Li et al. reported that the optimal grains size for Nd-Fe-B sintered magnets is 3.5–7 μm [42]. SEM images show that the grain sizes of the samples depend strongly on the annealing time, and as a profound consequence, the coercivity also depends on the annealing time. Sample A milled for 8 h exhibits grain sizes mainly in the range of 2–6 μm after annealing at 1123 K; this is a rather optimum annealing time in this case (Figure 4 Right). However, as was shown in this figure, there are still grains smaller than 2 µm and larger than 6 µm for the applied experimental conditions, which would explain the moderate coercivity of the annealed samples. The powder of sample A milled for 4 h after annealing at 1123 K contains a large variation of grain size from 2–4 μm to 20 μm or larger (Figure 4 Left), and such a wide range of grain sizes is not optimal for increasing the coercivity, as was evident from SEM images.

**Figure 5:**
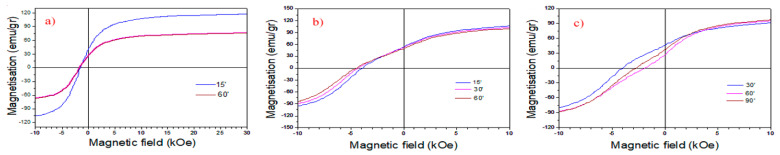
Demagnetization curves of annealed SmFe_9_Co_2_Ti powders at 973 K (**a**), 1073 K (**b**), and 1173 K (**c**); all samples were milled during 4 h.

In Figure 5, the demagnetization curves of sample A milled for 4 h and annealed at different intervals of time (15–90 min) and temperature range 973 K–1173 K are presented. The coercivity reached the maximum value of 4.6 kOe (0.46 T μ_0_H_C_) for the sample annealed at 1073 K for 60 min, while the magnetization decreased from 123 Am^2^/kg for the sample annealed during 15 min to 118 Am^2^/kg for annealing for 60 min. The SmFe_9_Co_2_Ti samples annealed at 1173 K exhibited a higher amount of α-(Fe, Co) than when annealed at 1073 K, and this can explain their moderate coercivity values, which reached a maximum of 4.04 kOe (0.40 T μ_0_H_C_) after annealing for 30 min.

**Figure 6:**
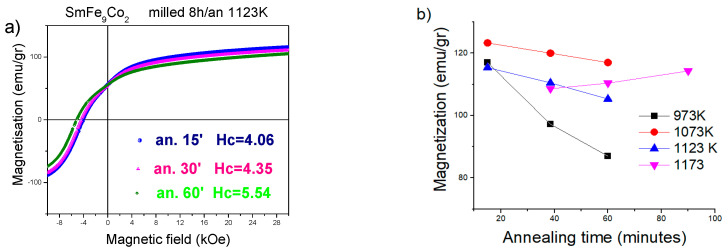
(**a**) Demagnetization curves SmFe_9_Co_2_Ti powders milled 8 h and annealed at 1123 K; (**b**) the respective magnetization values at 3T of sample annealed at 973 K (black), 1073 K (red), 1173 K (magenta) (milled 4 h), and 1123 K (blue) (milled 8 h).

Figure 6a presents the demagnetization curves of sample A annealed at 1123 K, measured up to a maximum field of μ_0_H = 3 T. The specific mass magnetization at the maximum external field decreased slightly from 115 Am^2^/kg (annealing 15 min) to 105 Am^2^/kg (annealing time 60 min). The trend of magnetization for the sample A at 973 K, 1073 K, 1173 K (milling time 4 h), and 1123 K (milling time 8 h) is depicted in Figure 6b. The magnetization acquired values in the range between 105 and 123 Am^2^/kg.

**Figure 7:**
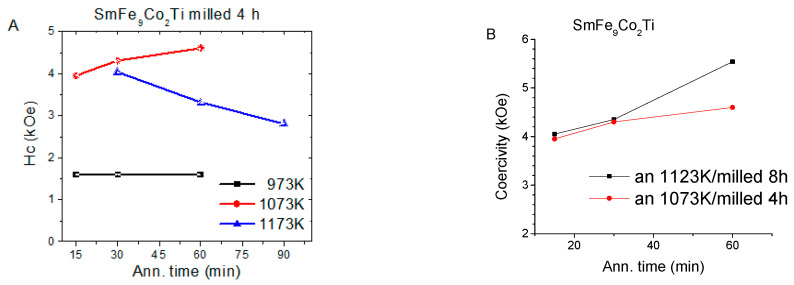
(**A**) Coercivity values of SmFe_9_Co_2_Ti powders milled for 4 h and annealed at 973 K, 1073 K, and 1123 K versus annealing time; (**B**) coercivity of SmFe_9_Co_2_Ti versus annealing time; the samples were milled for 4 and 8 h, respectively, at 1073 K and 1123 K.

The coercivity of sample A reached the highest maximum value of 5.5 kOe (0.55 T μ_0_H_C_) after annealing for 60 min (Figure 7B). However, this value was lower than expected due to the precipitation of the soft α-(Fe, Co) phases, as is evident in the X-ray diffraction plots (Figure 3b). Figure 7B shows the coercivity values of sample A milled for 4 and 8 h and then annealed at 1073 K and 1123 K, respectively. These results suggest that the optimal milling time is 8 h, and the optimal annealing temperature is 1123 K for our experiments. The importance of processing for coercivity is evident; in our study, we did follow conventional processing procedures that are compatible with common metallurgy practices, as these are the most commonly reported in the literature. However, it would be interesting to extend the study to other processing techniques like those reported in [43,44] for other systems.

**Figure 8:**
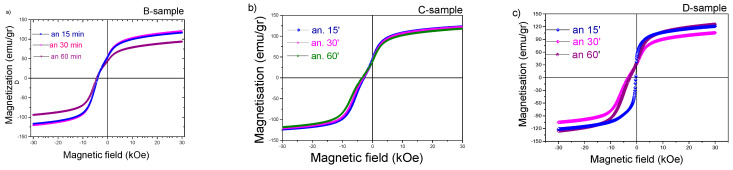
Demagnetization curves measured at 15, 30 and 60 minutes: (**a**) sample B (SmFe_9.04_Co_2.26_Ti_0.7_) annealed at 1123 K, (**b**) sample C (Sm_0.75_Zr_0.25_Fe_9_Co_2_Ti) annealed at 1098 K, and (**c**) sample D (Sm_0.75_Zr_0.25_Fe_9.04_Co_2.26_Ti_0.7_) annealed at 1123 K.

The demagnetization curves for the samples B, C, and D annealed at 1098 K and 1123 K are shown in the Figure 8. The coercivity was below 4 kOe (0.40 T μ_0_H_C_) for samples C and D where Sm was partially substituted by Zr; however, it improved to 4.3 kOe (0.46 T μ_0_H_C_) after annealing at 1123 K for 60 min. The expected decrease in magnetization after Zr substitution at samples D was compensated by the decrease in Ti content. In Table 1, the respective values of coercivity and magnetization are provided, and the value of magnetization were measured at 3 T, which is almost close to the saturation magnetization.

## 4. Conclusions

SmFe_12_-based alloys, stabilized by Ti and Zr, were prepared by arc melting under argon atmosphere, and we aimed to investigate the magnetic properties of their annealed HEBM powders. The arc-melting samples without Zr were almost single-phase 1:12 compounds with a small amount of a-(Fe, Co), while after substitution of Sm with Zr, a small quantity of Sm(FeCo)_2_ Laves-type phases appeared. After milling, the samples exhibited an amorphous/disordered phase, which was successfully recrystallized into a majority 1:12 phase after annealing. The milling time as well as the annealing process heavily affected the coercivity. The grain sizes of the samples milled for 8 h had a narrow distribution of 2–6 µm after annealing, while the grain sizes of the samples milled for 4 h had a wider distribution of 2–6 to 20 µm. As a result, the coercivity, as an external property closely related to the grain sizes, was higher for the annealed samples preliminarily milled for 8 h. A coercivity of μ_0_Hc = 0.55 T was obtained for the sample A milled 8 h and annealed at 1123 K. However, there were still large grains smaller than 2 μm and larger than >6 μm for the applied experimental conditions, which would explain the moderate coercivity of the annealed samples. The coercivity decreased after substitution of Sm for Zr due to precipitation of a third Laves-type phase and possible traces of other phases. The small amount of Sm-oxide also affects the coercivity in a negative direction. The magnetic properties of specific materials are promising for applications, considering the availability, the criticality, and the cost of the raw materials. The mass magnetization is considerably large, but more study on the improvement of coercivity is needed. This trade-off is evident when comparing, for example, the Sm_8_Fe_75.5_Ga_0.5_Ti_8_V_8_ materials mentioned before: our values of coercivity were almost half, but the larger magnetization and the absence of expensive materials like Ga provide potential. Homogenization of grain size in the material is important; however, the largest barrier in achieving practical bulk coercivity is the processing part. In R-T intermetallic compounds, the coercivity usually arises from R-rich phases at the grain boundaries of the final product, and up to now, no such phase has emerged. Such a phase, especially magnetic, could pin the movement of the domain walls of the material and improve the coercivity. It is evident that more work is needed in the mechanical processing of 1:12 alloys to achieve higher coercivity values.

## Figures and Tables

**Table 1:** Coercivity and magnetization values of samples B, C, and D.

	Sample B Ann. at 1123 K		Sample C Ann. at 1098 K		Sample D Ann. at 1123 K	
Ann. time	Hc (kOe)	M_3T_ (emu/gr)	Hc (kOe)	M_3T_ (emu/gr)	Hc (kOe)	M_3T_ (emu/gr)
15 m	4.01	120.9	3.0	122.8	4.05	120.8
30 min	4.05	120.7	3.1	122.1	3.29	105.5
60 min	4.37	93.6	3.5	118.4	2.76	125.6

## Data Availability

The original contributions presented in this study are included in the article. Further inquiries can be directed to the corresponding author.
